# Risk factors of in-home unintentional injuries among 0-6-year-old children in Changsha city of China: a cross-sectional survey based on Bronfenbrenner’s ecological system theory

**DOI:** 10.1186/s12887-022-03661-z

**Published:** 2022-10-17

**Authors:** Yihan Ma, Juan Song, Ming Hu, Rusi Yang, Panzi Yang

**Affiliations:** Department of Epidemiology and Health Statistics, Xiangya School of Public Health, Central South University, 110 Xiangya Rd., Changsha, 410078 China

**Keywords:** In-home unintentional injury, Risky behavior, Parental supervision, In-home environment, Children

## Abstract

**Background:**

In-home unintentional injuries (IUIs) seriously threatened children’s safety. Three factors, including risky behaviors, parental supervision, and home environmental risks, have been identified as major causes for IUIs. Studies considering the interrelations between the three were limited and no relative studies has been carried out among Chinese children. The purpose of this study is to fully explore the influences of behavioral, supervisory and environmental risk factors on IUIs and their associations among Chinese children on the bases of our self-developed scales.

**Methods:**

Through stratified cluster sampling, a cross-sectional survey was conducted with 798 parents of children aged 0 ~ 6 years in Changsha, China. Social demographics and IUIs history in the past year were collected by self-administered questionnaires. Three IUI-related scales, which had been developed and validated by our team, aimed to measure risks from children behavior, parental supervision and in-home environment. Structural equation models were constructed to analyze the relationship of these factors and their influences on IUIs using SPSS 26.0 and AMOS 22.0.

**Results:**

Seven hundred ninety-eight parents were surveyed in total, and 33.58% of them reported with IUIs history of their children. *X*^2^/df, goodness-of-fit index (GFI), adjusted goodness-of-fit index (AGFI) and the root-mean-square error of approximation (RMSEA) for the model of the whole children were 4.832, 0.879, 0.856 and 0.069 respectively, indicating an acceptable level of model fit. Direct influences were discovered between risky behaviors and children’s IUIs. Home environmental risks indirectly exerted impacts on IUIs by the mediating effect of risky behaviors, while the significant effect of parental supervision only existed in children aged 4-6 and girls.

**Conclusions:**

Risky behaviors played a mediating role in IUIs among children. Supervision and environmental risks affected IUIs indirectly by the exposure to risky behaviors. Parental supervision may not be able to offset the risks posed by the environmental and behavioral factors, so effective IUIs prevention strategies should focus on behavioral and environmental interventions, with appropriate supervision strategies based on the age and sex characteristics of the child.

**Supplementary Information:**

The online version contains supplementary material available at 10.1186/s12887-022-03661-z.

## Background

Childhood unintentional injuries (UIs) have been a significant public health problem worldwide. According to the Global Burden of Diseases report [1], more than 200 thousand children’s death occurred due to UIs each year across the global, and at least half of these injuries occurred at home. In-home unintentional injuries (IUIs) are major contributors to the global disease burden. It was reported that IUIs were responsible for 12.49% mortality and nearly 1.9 million Disability-Adjusted Life Years (DALYs) of Chinese children and adolescents (aged 0 to14 years) in 2019 [2]. Moreover, younger children (0 ~ 6 years), who lack adequate self-protective abilities and comprehensive understanding of surrounding conditions, thus their IUIs are more severe comparing with other age groups [3]. Hazards of child IUIs come from many sources, from the individual to the environmental level, which makes it too complex to adequately understand IUIs at a single level. To provide important reference for injury prevention, exploration of the associations between risk factors of IUIs is essential.

**Fig. 1:**
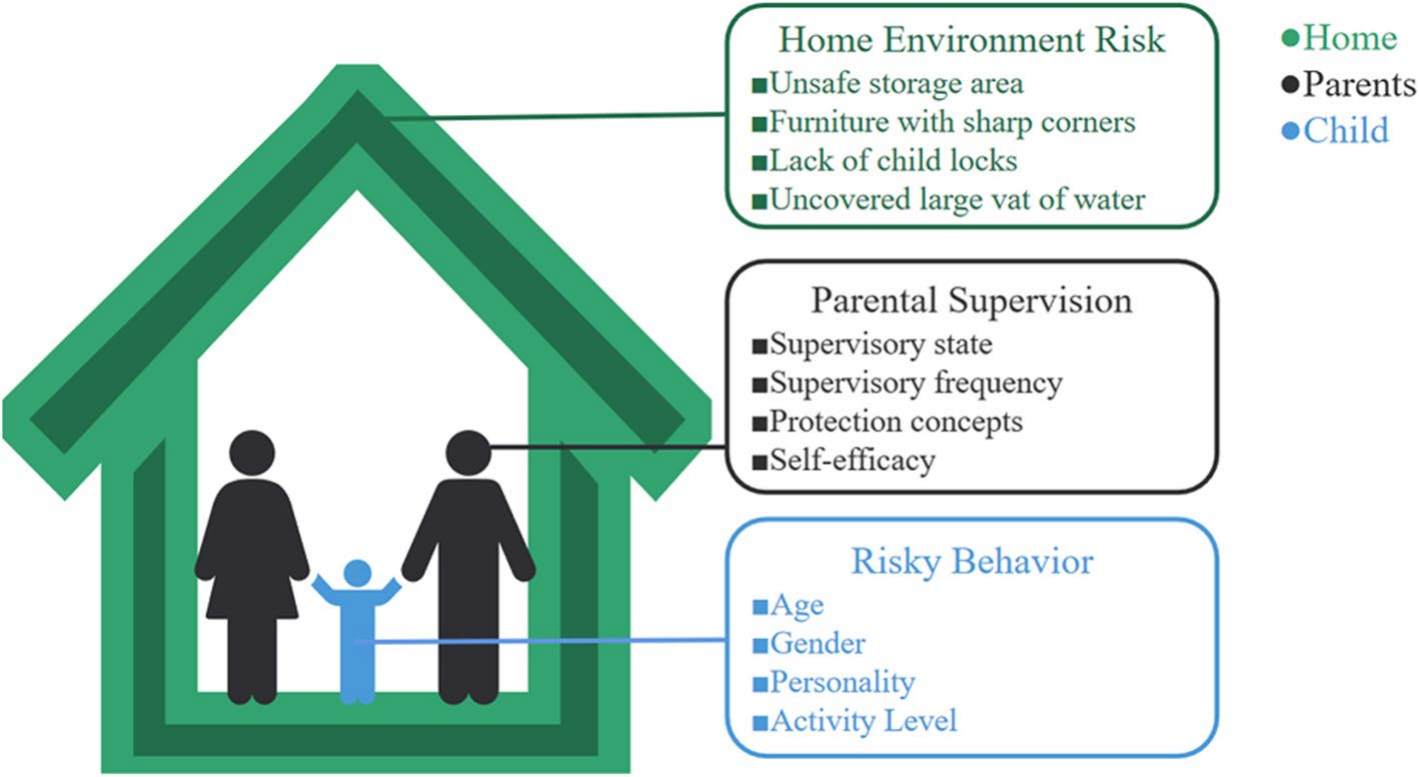
An ecological model of influencing factors of IUIs among children

Bronfenbrenner’s ecological theory contributes to understand children’s IUIs and the ever-changing relation with risk factors, in which assumes that research on child development emphasizes the contextuality of development and the complex interactions between children and their living environment [4]. It subdivides the environment into multiple levels (e.g., microsystems, mediating systems, exosystems, and macrosystems), each of which contains factors that can facilitate, allow, or inhibit children’s performance in that system. Most closely associated with IUIs for 0 to 6 years is the household ecosystem, as shown in Fig. 1. At the core is each child, where individual behavioral characteristics would certainly influence the course of IUIs. Regarding behavioral factors, studies reported numerous injuries while children engaging in risky behaviors [5–7]. Also, due to the aggregation effect, the impact of risky behavior on IUIs may be doubled and redoubled [7]. Previous studies shed light on the role of gender, temperament, personality and cognitive development to injury-related behaviors. They emphasized that boys, aggression, impulsivity, sensation seeking and mental disorder can promote children to adopt more frequent or serious behaviors [6, 8, 9], which suggested that risky behavior played a special role in childhood UIs. Parental supervision is another decisive factor to child IUIs. Past studies [10–12] indicated that parental supervision can counteract the potential injury hazard. In the presence of high-quality adult supervision, injuries may occur at a lower rate, although the effect is split between parents [13, 14]. Moreover, reports from Morrongiello [5, 7] revealed that parents would differ greatly in their actual supervisory performance when driven by inconsistent risk perception and safety knowledge. Thus, the protective effect of parental supervision was not cast in stone and may be influenced by other factors. Home environment is an important enabler in macro level of the household ecosystem. Children exposed to domestic factors are more vulnerable to IUIs, such as open-air burning coal, absence of balcony guardrail, lack of safe storage area and etc. [15, 16]. Historically, child IUIs programs have largely reduced risk to children by improving the home environment. A systematic evaluation [17] demonstrated the effectiveness of environmental change interventions in reducing the risk of IUIs. In addition, the combined effects of different factors on the IUIs occurrence had been observed. Zhou [18] and Zhang [19] have reported that higher levels of parental knowledge, attitudes and kills on supervising predicted lower injury risks when children were engaging in unusual behavior. Other studies [20, 21] on child injuries in farms have found that disquieting behavior for children aged 0 ~ 6 was more likely to lead to injury in high-risk environments. However, the existing studies still have some limitations: on the one hand, foreign studies could not be well derived to China to represent the IUIs situation of Chinese children because of the cultural diversity and regional disparity; on the other hand, associations that between the IUI-related behaviors, supervision and environmental risks have not been fully explored yet.

To fill the knowledge gap, we used structural equation model (SEM) to statistically analyze the variables that we interested in and to further explore the associations between the three factors under the ecological system above. Measurement tools for the present study stems largely from our previous research program, in which scales for injury-related risky behaviors, parental supervision and home environment risks of Chinese children have been developed and validated. Findings of this study along with other information might may help to provide a scientific basis for targeted IUIs prevention and control among children aged 0 ~ 6.

## Methods

### Participants

This study was a cross-sectional study conducted from October to November in 2017 and the subjects were children aged 0 ~ 6 and their families in the urban area of Changsha. According to the gross national product of each district in Hunan province in 2015, six administrative districts in Changsha are divided into two layers (layer 1: Furong district, Yuhua district and Tianxin district; layer 2: Kaifu district, Yuelu district and Wangcheng district). In each district, community health service centers (children under 3) and kindergartens (3-6-year-old children) served as research site to recruit participants, consistent with our previous studies under this program [22–24].

### Data collection

Questionnaires were completed by the primary caregiver of children, which defined as the person who spent most of the time at home on care of children. For ease of understanding, they are referred to as “parents” in this study. Non-primary caregivers who lived with the child and can accurately recall the occurrence and care of the child’s IUIs in the past year were also eligible to participate in this survey.

The investigators, who were all postgraduate students from Central South University, were trained prior to the survey. We obtained consent from the relevant heads of community health service centers and kindergartens for on-site support and organizational coordination. To reduce reporting bias, targeted data collection methods were used. At community health service centers, after detailed explanation by the investigator, questionnaires would be filled out by parents on their own or by investigators asking parents directly for accurate completion; in kindergartens, the teachers of the study site classes have been informed of the rules for our questionnaires in advance, and utilized the parent-teacher conferences to assist the investigators in requiring parents to complete the questionnaire independently. Double entry verification was used to assess the accuracy, logic and completeness of the data; questionnaires completed over 80% were considered valid and complemented with the median or mean.

### Measuring instruments

Demographic information assessed the characteristics children (age, gender, only child, residence) and their families (education level of parents, family structure and average family income).

Children’s risky behavior is operationally defined as a behavior that could lead children to engage in physically risky activities that result in injury when there are alternative behaviors that do not do so [25], here which was measured by the Children’s Risky Behavior Scale [24]. The tool consisted of 10 dimensions that children commonly encountered at home: animal bites, burns, falls, sharp instrument injuries, crush injuries, foreign body injuries, suffocation, poisoning, drowning and mechanical injuries. Each item referred to a separate activity or behavior that a child could potentially perform. The reliability of the total scale is high (Cronbach’s α coefficient = 0.94). The behavioral hazards were divided into low risk (≤66 points), medium risk (66 ~ 81 points) and high risk (≥81 points), taking percentile as the boundary value.

Parental supervision referred to a series of monitoring and tracking of children’s whereabouts, activities and adaptability adopted by primary caregivers [26], and was evaluated employing the Parental Supervision Trait Scale [23]. There were 7 dimensions (prevention concepts, fate, supervisory values, protection values, self-efficacy, supervisory frequency and supervisory states) with 27 items scored on a 5-point Likert scale. Cronbach’s α coefficient was 0.86, and the correlation coefficient between the seven dimensions and the total scale was between 0.053 and 0.679 (*P* < 0.001). The level of parental supervision ability was divided into three grades: lower parental supervision (≤110 points), medium parental supervision (111 ~ 119 points) and higher parental supervision (≥120 points).

The In-home Environmental Risks Scale [22] was performed to assess home environmental risk, which defined as the potential hazards at home that may cause IUIs and threaten children’s health [27]. It contains 6 dimensions which asks parents to identify environmental risks about falls, external force injuries, burns, poisonings, foreign body injuries and animal injuries. All items used a 5-point Likert scale response format. Cronbach’s α coefficient was 0.87, and the correlation coefficient between the six dimensions and the total scale was between 0.53 and 0.84 (*P* < 0.001). Higher scores indicate more internal risk of home and the environmental risk level could be divided into lower (< 71 points), medium (71 ~ 88 points) and higher (≥89 points) environmental risk.

According to the International Classification of Diseases, 10th edition [28] and the characteristics of children aged 0-6 years, the IUIs criteria were established: (a) children need to visit a medical unit and diagnose a certain type of injury; or (b) children need emergency treatment or care by family members; or (c) children has restricted activity or suspension of more than half a day. For those reported with IUIs, more specific injury registration cards were required to complete to fully explore the frequency and severity of injuries, which were measured respectively with one item (Number of child’s IUIs in the past year) and three items (Treatments of IUIs in the past year; Days of restricted movement due to IUIs; Days of treatment due to IUIs). All caregivers’ responses were converted into scores as a 5-point Likert scale.

This study was funded by the National Natural Science Foundation of China (No. 81402770) and approved by the ethics committee of the School of Xiangya Public Health at Central South University (Approval Number: CTXY-140002-4). All participants signed informed consent.

### Statistical analysis

We described respondents’ demographics with SPSS 26.0 and examined the direct and indirect relationship between latent constructs by the SEM of AMOS 22.0. Through item parceling procedure, dimensional variables were regarded as observation variables to estimate the corresponding construct with the average scores of assembled items, as it can maintain parsimony and stable estimates of the model [29]. Confirmatory factor analysis (CFA) was conducted initially to ascertain reliability and validity of the indicators used to operationalize the constructs. In the SEM analysis, we first proposed a hypothetical structure model and then modified through deleting non-significant paths and consulting modification indices. Maximum likelihood estimation was used with robust standard errors to provide better estimates and bootstrap ML methods were used to analyze the magnitude and significance of the direct, indirect and total effects of the constructs [30]. We created 3000 bootstrap samples (resampled from the original dataset) in order to derive Bias-corrected (BC) 95% confidence interval (CI) and Percentile 95%CI. Previous research [5, 13, 31] has shown that boys tend to take more risks than girls and preschoolers younger than 4 years were at higher risk of injury than older ones. Accordingly, multi-sample analysis was then conducted for examined variance of path coefficients, after we established the model of the general sample with an adequate fit.

## Results

### General information

A total of 845 participants were involved in and the valid questionnaires were 798 with the response rate of 94.44%. The mean age for children participated in was 3.357 (SD 1.677). 268 (33.58%) reported with IUI history in the past year and 52 participants (6.52%) had at least 2 IUIs. The mean of IUIs was 0.400 (*SD* = 0.681). Other social demographic data are presented in Table 1.

**Table 1:** Distribution of 0-6-year-old children and their families (n, %)

Characteristics	With IUIs history	Without IUIs history	*X*^2^	*P*
Gender			4.785	0.029
Male	150(35.63)	271(64.37)		
Female	107(28.38)	270(71.62)		
Age (years)			13.646	< 0.001
0 ~ 3	88(24.79)	267(75.21)		
4 ~ 6	164(37.02)	279(62.98)		
Only child			0.085	0.770
Yes	144(31.17)	318(68.83)		
No	108(32.14)	228(67.86)		
Resident status			0.045	0.832
Local	169(31.83)	362(68.17)		
Migrant	83(31.09)	184(68.91)		
Separate room			14.605	< 0.001
Yes	158(37.53)	263(62.47)		
No	94(24.93)	283(75.07)		
Education level of father			0.585	0.444
High School or below	133(30.43)	304(69.57)		
Bachelor degree or above	119(32.96)	242(67.04)		
Education level of mother			0.012	0.904
High School or below	157(31.72)	338(68.28)		
Bachelor degree or above	95(31.35)	208(68.65)		
Family structure				
Nuclear family	130(32.99)	264(67.01)	1.479^a^	0.773
Stem family	119(30.13)	276(69.87)		
Single-parent family	0(0.00)	1(100.00)		
Joint family	3(37.50)	5(62.50)		
Average family income			2.806	0.422
AFI < 5000RMB	86(34.54)	163(65.46)		
5000 ≤ AFI < 8000RMB	64(27.59)	168(72.41)		
8000 ≤ AFI < 10000RMB	35(31.53)	76(68.47)		
> 10000RMB	67(32.52)	139(67.48)		

^a^Fisher’s exact test

### Confirmatory factor analysis

Through item parceling procedure, IUIs, risky behaviors, parental supervision and home environmental risks were measured by the dimensions of each scale; and these constructs thus, adopted 4, 10, 7 and 6 indicators as observational variables respectively. All factor loadings were statistically significant (*P* < 0.05), ranging from 0.384 to 0.959 (see Supplementary Table 1, Additional file 1). All latent variables the composite reliabilities were higher than 0.8 and the average variance extracted (AVE) of each construct was greater than its squared correlations with other constructs. Next, the square roots of all AVEs were above 0.6 (see Supplementary Table 2, Additional file 1). The fit indices GFI, AGFI, CFI, NFI, RMR and RMSEA for each latent variable were acceptable and specific results were shown more in detail (see Supplementary Table 3, Additional file 1).

### Structural equation model

**Fig. 2:**
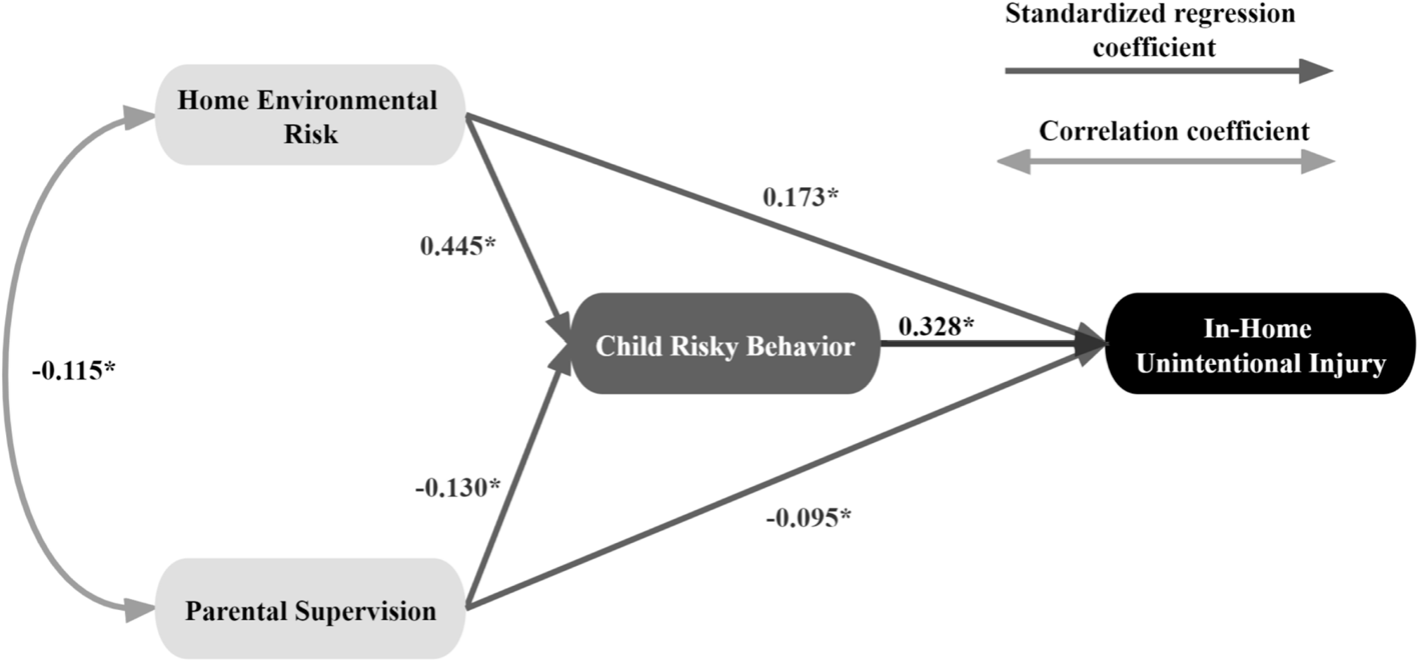
SEM of Chinese children’s risky behavior, parental supervision, home environmental risks and IUIs. Notes: * indicates that the path estimate is significant (*P* < 0.05)

Referring to previous experience, we first proposed the IUIs model for all children aged 0 ~ 6. The fit indices of the model are displayed in Table 2, showing an acceptable goodness-of-fit, in line with recommendations by Doll [32]. Parental supervision had negative effects on IUIs (*Unstd.* = − 0.083, *Std. =* − 0.095); home environmental risk (*Unstd. =* 0.202, *Std. =* 0.173) as well as risky behaviors (*Unstd. =* 0.243, *Std. =* 0.328) had positive effects on IUIs; and risky behaviors was affected by parental supervision (*Unstd. =* − 0.153, *Std. =* − 0.130) and home environmental risk (*Unstd. =* 0.705, *Std. =* 0.445). All path coefficients above were statistically significant, as shown in Fig. 2 and Table 3. Relationships involving potential mediation effects were discovered (Table 2). A statistically significant mediated effect existed from parental supervision to IUIs through risky behaviors ((*Unstd. =* − 0.037, *Std. =* − 0.043, BC 95% *CI*: − 0.067 ~ − 0.017, Percentile 95% *CI*:-0.065 ~ − 0.015). Similarly, the home environmental risks via children’s risky behavior had an indirect effect on IUIs (*Unstd. =* 0.171, *Std. =* 0.146, BC 95% *CI*:0.113 ~ 0.253, Percentile 95% *CI*: 0.111 ~ 0.250). The total effect of risky behaviors on IUIs was 0.328, higher than that of home environmental risks (*Unstd. =* 0.373, *Std. =* 0.319) and parental supervision (*Unstd. =* − 0.120, *Std. =* − 0.137). The specific data are shown in Additional file (see Supplementary Table 4, Additional file 1).

**Table 2:** Fit indices for the structural equation models

Model	*X*^2^/df	GFI	AGFI	CFI	RMR	RMSEA
General children	4.832	0.879	0.856	0.878	0.030	0.069
Children aged 0 ~ 3	2.983	0.846	0.817	0.868	0.028	0.075
Children aged 4 ~ 6	3.028	0.868	0.843	0.879	0.034	0.068
Boys	3.067	0.857	0.830	0.871	0.032	0.070
Girls	3.007	0.848	0.819	0.873	0.031	0.073

**Table 3:** Path coefficients between latent variables

Path	Parameter estimation					*CR*
	*Unstd.*	*Std.*	*S.E.*	*t*	*P*	
Home environmental risk →Risky behavior	0.705	0.445	0.069	10.217	*	10.171
Parental supervision →Risky behavior	−0.153	−0.130	0.043	−3.558	*	−3.568
Risky behavior →IUI	0.243	0.328	0.034	7.147	*	7.043
Home environmental risk →IUI	0.202	0.173	0.051	3.961	*	3.990
Parental supervision →IUI	−0.083	− 0.095	0.031	−2.677	0.009	−2.630

*Unstd.* Unstandardized estimates, *Std.* Standardized estimates

*indicates that the path estimate is significant (*P* < 0.05)

To check whether the associations between factors were equally present in different subgroups, we again validated them for different gender and age groups of the population. Models in each subgroup were fitted in an acceptable way (Table 2). The *X*^2^ values of the model comparisons between the different gender groups (*X*^2^ = 19.462, *P* = 0.002) and age groups (*X*^2^ = 29.005, *P* < 0.001) were significant, suggesting that regression weights of among subgroups were variant. Children aged 4 ~ 6 (*Unstd. =* 0.282, *Std. =* 0.426) and boys (*Unstd. =* 0.281, *Std. =* 0.384) displayed greater behavioral effects than children aged 0 ~ 3 (*Unstd.* = 0.211, *Std.* = 0.231) and girls (*Unstd.* = 0.173, *Std.* = 0.232). For all subgroups, the effects of home environmental risks were statistically significant, among which environmental risks had greater indirect impacts on IUIs of children aged 4 ~ 6 (*Unstd. =* 0.397, *Std. =* 0.201) than that of children aged 0 ~ 3 (*Unstd. =* 0.095, *Std. =* 0.108). Further, the greater direct effects of environmental risks on IUIs were examined in girls (*Unstd. =* 0.299, *Std. =* 0.241) than in boys (*Unstd. =* 0.126, *Std. =* 0.115), but the indirect effect did the opposite. In terms of parental supervision, there were greater negative impacts found in children aged 4 ~ 6 (*Unstd. =* − 0.148, *Std. =* − 0.158) and girls (*Unstd. = −* 0.202, *Std. =* − 0.249) than children aged 0 ~ 3 (*Unstd. =* − 0.100, *Std. =* − 0.116) and boys (*Unstd. =* − 0.035, *Std. =* − 0.038), respectively. Except for boys, the effects of parental supervision proved statistical significance, as follows in Additional file (see Supplementary Table 4, Additional file 1).

## Discussions

In our study, the incidence of IUIs was 33.42%, higher than the results in other studies [15, 33], which may be the result that our subjects were children aged 0 ~ 6, who occupied a higher injury risk than other age groups. In addition, children aged 0 ~ 3 in this study were investigated by face-to-face interview, which made the data of younger children more reliable and had a certain influence on the incidence rate. Meanwhile, this finding confirmed the necessity of special attention and targeting preventions towards IUIs among Chinese urban children, which need to be carried out with adequate evidence bases. Thus, based on Bronfenbrenner’s ecosystem theory and our previous efforts, we systematically explored the causes of children’s IUIs in Chinese urban areas and revealed significant relationships among risky behaviors, parental supervision, home environmental risks and IUIs through SEM.

The CFA exhibited that all latent variables had good reliability and discriminant validity through the results of composite reliabilities and the AVEs of each construct. GFI, CFI and RMSEA were all acceptable, so we considered that the measurement tools used in this study met the conditions for the structural model. Results of SEM of the general sample revealed that the total value of effect from risky behavior was much higher when compared with parental supervision and home environmental risks, though behavior was influenced by the latter two to some extent. Home environmental risks played direct and indirect roles in children’s IUIs, whereas parental supervision could only influence special groups, such as children aged 4 ~ 6 years and girls.

Our investigation tool for risky behaviors involved animal bites, burns, falls, suffocation and many other injury types, all of which corresponded to the relevant child IUIs, for example, the behavior of a child poking a power socket was in corresponding to the unintentional burns. SEM of subgroups showed the contribution of risky behaviors with aging, because the better motor control and coordination of older children, the higher possibility for hazard situations [34], which was similar to the fact that children who climb frequently were vulnerable to falls. Likewise, the behavioral effect was greater for boys than for girls, which may be due to the gender differences in early developmental trajectories for emotional expression. Boys are more inclined to express anger-related emotions and have greater tendency to engage in rough and tumble play than girls [35], which leads to more frequent conduct problems and more serious IUIs. Similarly, multimethod strategies study [36] revealed that most of the boy’s injuries followed misbehaviour (i.e., mothers reported that 60% of boys suffered significantly more injuries when they engaged in inappropriate behavior). Also, boys express more optimistic bias and tend to attribute injuries to bad luck, while girls express more considerations about the consequences of their actions in the same situation [37]. Thus, our results served as reminders for IUIs prevention programs, in which appropriate efforts according to age and gender characteristics may be needed.

Despite risky behaviors occupied the strongest effect on children’s IUIs, reducing risks caused by risky behaviors required more reasonable parental supervision and better home environment. The direct effects of environmental risks and supervision were validated across the entire sample, similar to previous studies [33, 38]. More importantly, we proved the indirect role of the both on IUIs. The underlying mechanism may be that parents who provided direct attention, with closest proximity on a continual basis were able to moderate their children’s injury-prone tendencies and ensure child safety in the home setting [39], and home environments may influence the emergence of externalized behavior problems in children and thus create favorable conditions for being exposed to IUIs, in line with other studies [40, 41].

The indirect effects of the home environmental risks implied that the hazards of IUIs were on the rise when children with high behavioral risks are in unsafe households, particularly 4-6-year-old children and boys. The reason may be that high-risk settings would stimulate children to show more externalized behavioral problems and fostered interactions with hazards. Similarly, Schwebel [42] revealed that in a complicated housing environment, children with higher behavioral risk might judge environmental hazards with a rushed, impulsive manner, which might lead to misestimation of the risk involved in a particular activity, and in turn lead to injury. Therefore, the co-existence of direct and indirect paths highlighted the dual value of addressing risk factors in house. However, contrary to previous results, the direct impact of home risk varied more in girls than boys. A possibility involved greater perceptual sensitivity in girls. If girls are more attuned to the fine details of their environment, they will experience more stressors and curiosity, which can trigger their susceptibility to more injury events around them [43]. Also, this finding suggested that injury risk for girls arose more from poor conditions that already existed at home than motivation of child’s behaviors from home environment, while the opposite was true for boys. Hence, reducing IUIs risk in girls necessitates more complete disengagement from dangerous situations, and for boys, timely home inspection and regulating children’s behavioral tendencies may be more important.

Results suggested that parental supervision was not significant to deter younger children’s injury risk, either directly or indirectly. We speculated that this may be associated with differences in child care. Morrongiello [44] reported that parents of younger children only focused on increasing the frequency and proximity of observations and routinely overestimate desired effect of their supervision strategies. By contrast, parents of older children exercised safety education and fear-inducing strategy, in which parents would stress the serious consequences if children did not follow their guidance. In addition, the early IUI experiences of 4-6-year-old children reminded their parents of the relative necessity for parental supervision, which made the roles of supervision reported by parents more prominent. We did not find any significant effects on parental supervision of boys, but did of girls, which was beyond our expectations. One possible explanation was that specific societal expectations for males and females. Boys are always expected to protect their families and to overcome dangers that interfere with their ability to provide for their families, whereas girls are allowed to show more emotions to take on the traditional role of caregivers [35]. Thus, more encouragements to participate in risky games are given to boys, while more caution about safety to girls, which relatively weakened the roles of supervision.

Whether in the general population or each subgroup, risky behavior was involved as an intermediary, and meanwhile, the effects of risky behavior and home environment were greater than that of parental supervision, which made we acknowledge the relative materiality of all three in preventing IUIs. Although parental supervision has been advocated as a protective method in the past, we found that it separately cannot completely offset the crisis caused by environmental risks and behaviors with limited power. Consequently, the best strategy to moderate risk for IUIs is an effective combination of behavioral regulations and environmental interventions, and measures targeting parental supervision are considered as promising remedies. For younger children, this means keeping the consistency of space as much as possible to timely take behavioral modification and reasonable planning of indoor activity space. For older children with increased cognitive awareness, this signified necessity of regular health education and improvement of risk perception. Besides, specific efforts, such as caregivers’ safety education and enhanced safety equipment (e.g., child-resistant container, cabinet latches, stair gates), will be better able to contain crises from environment and supervision. In terms of gender, girls are highly sensitive to environmental risks, so it is critical to carry out a thorough screening of the risk of home from the perspective of children. At the same time, moderate behavioral constraints and verbal reminders may yield better results, because girls are more compliant with their parents [35]. For boys, efforts should be directed towards positive regulation of impulsive behavioral traits. Traditional didactic education should also be changed and more participatory activities can be adopted, such as training tasks in hazard perception, sound decision-making, and safety rules, which will be better targeted to ameliorate boys’ potential risks.

To decrease child IUIs more efficiently, more efforts need to be done besides the above measures. Current data of Chinese children’s IUIs stemmed mainly from the China’s Disease Surveillance Points system and National Injury Surveillance System consisted of 127 sentinel hospitals [45]; but due to the relatively low proportion of deaths and hospital visits caused by injury events, IUIs disposed of by family members were often missed. It is a call to attach importance to the collection, analysis and utilization of basic IUIs information. Measures to expand the monitoring sites to primary units (e.g., schools, kindergartens, and communities), standardize the information collection process, and formulate multi-sector strategies involving the whole society will be good complement to Chinese’s non-medical treatment IUIs. Moreover, parents in Chinese urban areas generally lacked sufficient recognition of the IUIs’ controllability, and especially parents who belong to floating families were basically in the blind spot of child IUIs prevention [46]. Therefore, further strengthening investment in home safety publicity, focusing on children with weaker parental supervision and larger needs of injury prevention, such as migrant children who often be left at home alone, urging parents to enhance literacy of IUIs may be the key to future injury control program.

### Limitations

There were still limitations in this study. Firstly, recall bias may be inevitable, as the study mostly was based on parents’ recollections. Secondly, the cross-sectional design limits the possibility of establishing causal relationships and can only explain the associations between factors. Thirdly, we did not investigate the influence of other potentially important factors, such as experience for past injuries and interventions from communities and kindergartens, were not available, and these factors might affect the outcomes as potential confounders. Fourthly, we verified direct and indirect associations between the factors, but the results suggested significant differences between the models for age and gender groups. The specific source of the differences and the reasons for their generation deserve consideration when explored in a larger sample. Finally, the disparities in IUIs between different cities and rural-urban areas cannot be ignored. Our inference, including what we described as mediation, relied on statistical indicators of model fit. Therefore, the model was an attempt for preliminary exploration. Further verification and enrichment on populations of different regions and prospective studies may be needed.

## Conclusion

Based on Bronfenbrenner’s ecological system theory, the present study revealed relations between risky behaviors, parental supervision, home environmental risks and IUIs among 0-6-year-old children in Chinese urban areas. The direct effects of the all three was demonstrated on IUIs of all children aged 0 ~ 6. Through the mediator of risky behaviors, parental supervision and home environmental risks indirectly influenced hazards of IUIs, although which varied by ages and genders. The results could provide favorable reference for targeting interventions to cut down IUIs’ rates and improve the quality of life of 0-6-year-old children. For children aged 0 ~ 3, strengthening behavior guidance and safe spatial planning is essential. With the growth of age, children’s IUIs prevention may call for a perfect combination of enhanced safety education, increased awareness of parental supervision and effective home safety equipment. Further, parental supervision of IUI is weak in boys, and efforts to improve their impulsive behavior characteristics are more important, while for girls, it is more urgent to eliminate environmental risks across the board.

## Supplementary Information

**Additional file 1: Supplementary Table 1.** Standardized factor loading for latent variables in the measurement model. **Supplementary Table 2.** AVE, correlations, and squared correlations of the latent variables. **Supplementary Table 3.** Fit values of each latent variable in the measurement model. **Supplementary Table 4.** The indirect, direct, and total effects of parental supervision and home environmental risks on IUIs.

## Data Availability

The datasets generated and/or analyzed during the current study are not publicly available due to privacy and confidentiality agreements but are available from the corresponding author on reasonable request.

## Abbreviations

UIs: Unintentional Injuries
IUIs: In-home Unintentional Injuries
CFI: Comparative Fit Index
GFI: Goodness-of-Fit Index
AGFI: Adjusted Goodness-of-Fit Index
NFI: Normed Fit Index
RMR: Root Mean Square Residual
RMSEA: Root-Mean-Square Error of Approximation
DALYs: Disability-Adjusted Life Years
SEM: Structural Equation Model
CFA: Confirmatory Factor Analysis
AVE: Average Variance Extracted

## References

[1] Institute for Health Metrics and Evaluation. Global burden of disease (GBD) results: University of Washington: Population Health Building/Hans Rosling Center. https://www.healthdata.org/gbd/2019. Accessed 10 Apr 2022

[2] Vos T, Lim SS, Abbafati C, Abbas KM, Abbasi M, Abbasifard M (2020). Global burden of 369 diseases and injuries in 204 countries and territories, 1990–2019: a systematic analysis for the global burden of disease study 2019. Lancet.

[3] World Health Organization. World health statistics 2015. World Health Organization; Geneva; 2015.

[4] Zhou N, Cheah CSL (2015). Ecological risk model of childhood obesity in Chinese immigrant children. Appetite.

[5] Morrongiello BA (2018). Preventing unintentional injuries to young children in the home: understanding and influencing parents’ safety practices. Child Develop Perspect.

[6] Morrongiello BA, Lasenby J (2006). Finding the daredevils: development of a sensation seeking scale for children that is relevant to physical risk taking. Accid Anal Prev.

[7] Morrongiello BA, Kiriakou S (2004). Mothers’ home-safety practices for preventing six types of childhood injuries: what do they do, and why?. J Pediatr Psychol.

[8] Garzon DL (2005). Contributing factors to preschool unintentional injury. J Dev Behav Pediatr.

[9] Schwebel DC, Gaines J (2007). Pediatric unintentional injury: behavioral risk factors and implications for prevention. J Dev Behav Pediatr.

[10] Wells M, Morrongiello BA, Kane A (2012). Unintentional injury risk in school-age children: examining interrelations between parent and child factors. J Appl Dev Psychol.

[11] Schwebel DC, Brezausek CM, Ramey SL, Ramey CT (2004). Interactions between child behavior patterns and parenting: implications for children's unintentional injury risk. J Pediatr Psychol.

[12] Schnitzer P, Dowd M, Morrongiello B, Kruse R (2015). Supervision and risk of unintentional injury in young children. Inj Prev.

[13] Morrongiello BA, Corbett M, Lasenby J, Johnston N, McCourt M (2006). Factors influencing young children’s risk of unintentional injury: parenting style and strategies for teaching about home safety. J Appl Dev Psychol.

[14] Ochi M, Fujiwara T (2021). Paternal childcare in early childhood and problematic behavior in children: a population-based prospective study in Japan. BMC Pediatr.

[15] Nooyi SC, Sonaliya KN, Dhingra B, Roy RN, Indumathy P, Soni RK (2021). Descriptive epidemiology of unintentional childhood injuries in India: an ICMR taskforce multisite study. Indian Pediatr.

[16] Munro SA, van Niekerk A, Seedat M (2006). Childhood unintentional injuries: the perceived impact of the environment, lack of supervision and child characteristics. Child Care Health Dev.

[17] Bhatta S, Mytton J, Deave T (2020). Environmental change interventions to prevent unintentional home injuries among children in low- and middle-income countries: a systematic review and meta-analysis. Child Care Health Dev.

[18] Zhou H, Fan L, Wu C, Luo A, Mo C, He G (2019). Understanding the associations among parents teaching safety rules to children, safety behaviors and unintentional injuries in Chinese preschool children. Prev Med.

[19] Zhang H, Bai F, Song H, Yang J, Wang X, Ye Q (2021). Cumulative effect of risk and protective factors on unintentional injury for Chinese rural children: a nested case-control study. BMC Public Health.

[20] Morrongiello BA, Marlenga B, Berg R, Linneman J, Pickett W (2007). A new approach to understanding pediatric farm injuries. Soc Sci Med.

[21] Morrongiello BA, Pickett W, Berg RL (2008). Adult supervision and pediatric injuries in the agricultural worksite - ScienceDirect. Accid Anal Prev.

[22] Wang Z, Ran Y, Nian H, Shao K, Yu T, Hu M (2020). Development and performance test of the environment scale of unintentional injury in the home for children aged 0-6 years old in urban area of China. Zhonghua Yufang Yixue Zazhi.

[23] Wan SQ, Nian HY, Yang J, Zhang BL, Hu M (2021). Development and reliability test of parental supervision scale for unintentional injury of children aged 0-6 in urban China. Chin J Health Stat.

[24] Song J, Shao K, Zhang BL, Wan SQ, Hu M (2021). Development and validation of risk behavior scale for in-home unintentional injury in urban children aged 0-6 years in China. Chin J Epidemiol.

[25] Morrongiello BA, Matheis S (2007). Understanding children’s injury-risk behaviors: the independent contributions of cognitions and emotions. J Pediatr Psychol.

[26] Dishion TJ, Mcmahon RJ (1998). Parental monitoring and the prevention of child and adolescent problem behavior: a conceptual and empirical formulation. Clin Child Fam Psychol Rev.

[27] Mayes S, Roberts MC, Stough CO (2014). Risk for household safety hazards: socioeconomic and sociodemographic factors. J Saf Res.

[28] World Health Organization. International classification of diseases.10th revision. Geneva; 2017. http://apps.who.int/classifications/icd10/browse/2010/en. Accessed 10 Apr 2022

[29] Hau KT, Marsh HW (2004). The use of item parcels in structural equation modelling: non-normal data and small sample sizes. Br J Math Stat Psychol.

[30] MacKinnon DP, Lockwood CM, Williams J (2004). Confidence limits for the indirect effect: distribution of the product and resampling methods. Multivariate Behav Res.

[31] Bernardo LM, Gardner MJ, Rosenfield RL, et al. A comparison of dog bite injuries in younger and older children treated in a pediatric emergency department[J]. Pediatr Emerg Care. 2002;18(3):247–9. 10.1097/00006565-200206000-00024 12066018

[32] Doll WJ, Xia W, Torkzadeh G. A confirmatory factor analysis of the end-user computing satisfaction instrument. MIS Q. 1994;18(4):453–61.

[33] Ma XQ, Zhang Q, Jiang R, Lu J, Wang HP, Xia QH (2021). Parents’ attitudes as mediators between knowledge and behaviours in unintentional injuries at home of children aged 0-3 in Shanghai, eastern China: a cross-sectional study. BMJ Open.

[34] Shi X, Shi J, Wheeler KK, Stallones L, Ameratunga S, Shakespeare T (2015). Unintentional injuries in children with disabilities: a systematic review and meta-analysis. Inj Epidemiol.

[35] Chaplin TM, Aldao A (2013). Gender differences in emotion expression in children: a meta-analytic review. Psychol Bull.

[36] Morrongiello BA, Ondejko L, Littlejohn A (2004). Understanding toddlers’ in-home injuries: I. Context, correlates, and determinants. J Pediatr Psychol.

[37] Morrongiello BA, Rennie H (1998). Why do boys engage in more risk taking than girls? The role of attributions, beliefs, and risk appraisals. J Pediatr Psychol.

[38] Drachler M, Leite J, Marshall T, Almaleh C, Feldens CA, Vitolo MR (2007). Effects of the home environment on unintentional domestic injuries and related health care attendance in infants. Acta Paediatr.

[39] Petrass L, Blitvich JD, Finch CF (2009). Parent/Caregiver supervision and child injury: a systematic review of critical dimensions for understanding this relationship. Fam Community Health.

[40] Khatlani K, Alonge O, Rahman A, Hoque DME, Bhuiyan A-A, Agrawal P (2017). Caregiver supervision practices and risk of childhood unintentional injury mortality in Bangladesh. Int J Environ Res Public Health.

[41] Morrongiello BA, Bryant L, Cox A (2021). Validation of a measure of injury-risk behaviors in the first 2 years of life: infant/toddler-injury behavior questionnaire (IT-IBQ). Infant Behav Dev.

[42] Schwebel DC, Plumert JM (1999). Longitudinal and concurrent relations among temperament, ability estimation, and injury proneness. Child Dev.

[43] Else-Quest NM, Hyde JS, Goldsmith HH, Van Hulle CA (2006). Gender differences in temperament: a meta-analysis. Psychol Bull.

[44] Morrongiello BA, Widdifield R, Munroe K, Zdzieborski D (2014). Parents teaching young children home safety rules: implications for childhood injury risk. J Appl Dev Psychol.

[45] Duan L, Deng X, Wang Y, Wu C, Jiang W, He S (2015). The national injury surveillance system in China: a six-year review. Injury.

[46] Xu T, Gong LM, Wang HS, Zhang R, Wang XY, Kaime-Atterhog W (2014). Epidemiology of unintentional injuries among children under six years old in floating and residential population in four communities in Beijing: a comparative study. Matern Child Health J.

