# Supplementary material for: Risk factors of in-home unintentional injuries among 0-6-year-old children in Changsha city of China: a cross-sectional survey based on Bronfenbrenner’s ecological system theory
Source: BMC Pediatr. 2022 Oct 17;22:598. doi: 10.1186/s12887-022-03661-z (PMC9575220; doi:10.1186/s12887-022-03661-z)
Supplement: Supplementary file 1 — Additional file 1: Supplementary Table 1. Standardized factor loading for latent variables in the measurement model. Supplementary Table 2. AVE, correlations, and squared correlations of the latent variables. Supplementary Table 3. Fit values of each latent variable in the measurement model. Supplementary Table 4. The indirect, direct, and total effects of parental supervision and home environmental risks on IUIs. [file 12887_2022_3661_MOESM1_ESM.pdf]

Supplementary materials

Supplementary Table 1. Standardized factor loading for latent variables in the measurement model

Supplementary Table 2. AVE, correlations, and squared correlations of the latent variables

Supplementary Table 3. Fit values of each latent variable in the measurement model

Supplementary Table 4. The indirect, direct, and total effects of parental supervision and home environmental risks on IUIs

Supplementary Table 1. Standardized factor loading for latent variables in the measurement model

Variables	Indicators	*S.E.*	Factor Loading	Composite Reliabilities
Risky Behavior	RB01	-	0.492	0.860
	RB02	0.098	0.715	
	RB03	0.231	0.711	
	RB04	0.112	0.655	
	RB05	0.174	0.802	
	RB06	0.076	0.588	
	RB07	0.120	0.721	
	RB08	0.094	0.384	
	RB09	0.181	0.643	
	RB10	0.062	0.395	
Parental Supervision	PS01	-	0.724	0.857
	PS02	0.063	0.502	
	PS03	0.052	0.614	
	PS04	0.041	0.959	
	PS05	0.049	0.462	
	PS06	0.073	0.708	
	PS07	0.060	0.729	
Home Environmental Risk	HER01	-	0.635	0.862
	HER02	0.066	0.836	
	HER03	0.077	0.727	
	HER04	0.084	0.688	
	HER05	0.098	0.704	
	HER06	0.070	0.685	
IUIs	Injury01	-	0.578	0.878
	Injury02	0.125	0.836	
	Injury03	0.067	0.848	
	Injury04	0.097	0.917	

Notes: RB01 to RB10 represents animal bites, burns, falls, sharp instrument injuries, crush injuries, foreign body injuries, suffocation, poisoning, drowning and mechanical injuries, respectively; PS01 to PS07represents prevention concepts, fate, supervisory values, protection values, self-efficacy, supervisory frequency and supervisory states, respectively; HER01 to HER06 represents environmental risks about falls, external

force injuries, burns, poisonings, foreign body injuries and animal injuries, respectively; Injury01 to Injury04 represents frequency of IUIs, treatment of IUIs, days of restricted movement, and days of treatment due to IUIs, respectively.

Supplementary Table 2. AVE, correlations, and squared correlations of the latent variables

	AVE	Risky Behavior	Parental Supervision	Home Environmental Risks	IUIs
Risky Behavior	0.391	**0.625**			
Parental Supervision	0.474	-0.176	**0.688**		
Home Environmental Risks	0.512	0.460	-0.113	**0.716**	
IUIs	0.648	0.425	-0.172	0.335	**0.805**

Supplementary Table 3. Fit values of each latent variable in the measurement model

Latent Variables	GFI	AGFI	CFI	RMR	NFI	RMSEA
Risky Behavior	0.958	0.934	0.946	0.008	0.934	0.072
Parental Supervision	0.948	0.843	0.919	0.050	0.916	0.162
Home Environmental Risk	0.960	0.907	0.955	0.008	0.950	0.111
IUIs	0.958	0.800	0.964	0.026	0.963	0.205

Supplementary Table 4. The indirect, direct, and total effects of parental supervision and home environmental risks on IUIs

Effects	Home environmental risk				Parental Supervision				Risky Behavior			
	Parameter		95% *CI*		Parameter		95% *CI*		Parameter		95% *CI*	
	Unstd.	*Std.*	Bias-corrected	Percentile	*Unstd.*	*Std.*	Bias-corrected	Percentile	*Unstd.*	*Std.*	Bias-corrected	Percentile
General children												
Indirect effects	0.171	0.146*	(0.113,0.253)	(0.111,0.250)	-0.037	-0.043*	(-0.067,-0.017)	(-0.065,-0.015)				
Direct effects	0.202	0.173*	(0.085,0.345)	(0.079,0.339)	-0.083	-0.095*	(-0.161,-0.020)	(-0.155,-0.015)				
Total effects	0.373	0.319*	(0.245,0.529)	(0.242,0.525)	-0.120	-0.137*	(-0.196,-0.055)	(-0.193,-0.052)	0.243	0.328*	(0.172,0.334)	(0.168,0.365)
Children aged 0~3												
Indirect effects	0.095	0.108*	(0.042,0.176)	(0.040,0.174)	-0.019	-0.022	(-0.056,0.001)	(-0.052,0.003)				
Direct effects	0.142	0.161*	(0.019,0.296)	(0.014,0.288)	-0.081	-0.093	(-0.184,0.011)	(-0.179,0.013)				
Total effects	0.237	0.269*	(0.111,0.398)	(0.107,0.388)	-0.100	-0.116*	(-0.199,-0.011)	(-0.197,-0.011)	0.221	0.231*	(0.096,0.367)	(0.095,0.364)
Children aged 4~6												
Indirect effects	0.397	0.201*	(0.218,0.681)	(0.212,0.670)	-0.036	-0.038	(-0.083,0.003)	(-0.079,0.009)				
Direct effects	0.425	0.215*	(0.153,0.800)	(0.150,0.794)	-0.112	-0.120*	(-0.219,-0.009)	(-0.222,-0.013)				
Total effects	0.821	0.416*	(0.512,0.327)	(0.503,1.310)	-0.148	-0.158*	(-0.262,-0.043)	(-0.261,-0.041)	0.282	0.464*	(0.288,0.548)	(0.289,0.549)
Boys												
Indirect effects	0.200	0.184*	(0.125,0.304)	(-0.037,0.038)	-0.001	-0.001	(0.118,0.293)	(-0.039,0.036)				
Direct effects	0.126	0.115*	(0.001,0.287)	(-0.002,0.280)	-0.034	-0.036	(-0.136,0.060)	(-0.136,0.060)				
Total effects	0.326	0.299*	(0.180,0.519)	(0.171,0.503)	-0.035	-0.038	(-0.147,0.061)	(-0.145,0.064)	0.282	0.384*	(0.277,0.485)	(0.272,0.480)
Girls												
Indirect effects	0.118	0.095*	(0.038,0.236)	(0.041,0.242)	-0.051	-0.063*	(-0.104,-0.019)	(-0.100,0.018)				

Direct effects	0.299	0.241*	(0.089,0.599)	(0.077,0.537)	-0.151	-0.186*	(-0.257,-0.065)	(-0.246,-0.056)								
Total effects	0.417	0.336*	(0.216,0.701)	(0.210,0.679)	-0.202	-0.249*	(-0.305,-0.129)	(-0.296,-0.120)	0.173	0.232*	(0.061,0.311)	(0.067,0.321)				
